# ‘De Winter’ electrocardiographic pattern in left bundle branch block and acute coronary occlusion: a case report

**DOI:** 10.1093/ehjcr/ytaf354

**Published:** 2025-07-24

**Authors:** Hugo Leonardi Baldisserotto, Andressa Daga, Guilherme Heiden Telo

**Affiliations:** Internal Medicine Department, Federal University of Rio Grande do Sul (UFRGS), Ramiro Barcelos, 2400, 90610-264 Porto Alegre/RS, Brazil; Cardiology Department, Hospital de Clínicas de Porto Alegre (HCPA), Ramiro Barcelos, 2350, 90035-003 Porto Alegre/RS, Brazil; Cardiology Department, Hospital de Clínicas de Porto Alegre (HCPA), Ramiro Barcelos, 2350, 90035-003 Porto Alegre/RS, Brazil

**Keywords:** De Winter pattern, Acute coronary occlusion, Left bundle branch block, Case report

## Abstract

**Background:**

ST-segment elevation on electrocardiogram (ECG) is traditionally interpreted as acute coronary occlusion, with a sensitivity of approximately 75%. Electrocardiographic patterns such as ‘Wellens’ and ‘De Winter’ have been studied as ST-segment elevation myocardial infarction (STEMI) equivalents.

**Case summary:**

A 79-year-old hypertensive woman was admitted with chest pain radiating to the neck. The initial ECG showed a left bundle branch block with a ‘De Winter’ pattern. After initial care, she underwent cardiac catheterization that revealed thrombotic occlusion of the left anterior descending artery. She underwent angioplasty with angiographic success and clinical improvement, leading to hospital discharge.

**Discussion:**

Recognition of electrocardiographic patterns associated with acute coronary occlusion is crucial for diagnosis and prompt reperfusion, reducing morbidity and mortality. The ‘De Winter’ pattern is an equivalent to STEMI. Patients with pre-existing conditions that affect QRS amplitude and width, such as the left bundle branch block, pose an additional challenge.

Learning points

- It is important to recognize the ‘De Winter’ pattern as a ST-segment elevation myocardial infarction (STEMI) equivalent, even in the presence of left bundle branch block (LBBB).
- Traditional STEMI diagnostic criteria in patients with LBBB are limited, making careful complementary evaluation essential.

## Introduction

Elevation of the ST-segment on the electrocardiogram (ECG) is classically interpreted as acute coronary occlusion; however, the sensitivity of this finding is around 75%.^1^ As a result, electrocardiographic patterns recognized as ST-segment elevation myocardial infarction (STEMI) equivalents have been increasingly studied, such as Wellens syndrome, the ‘De Winter’ pattern,^2^ and diffuse ST-segment depression associated with ST-segment elevation in aVR.^3^ We report here the case of a 79-year-old woman with left bundle branch block (LBBB) and a ‘De Winter’ pattern.

## Summary figure

**Figure:**
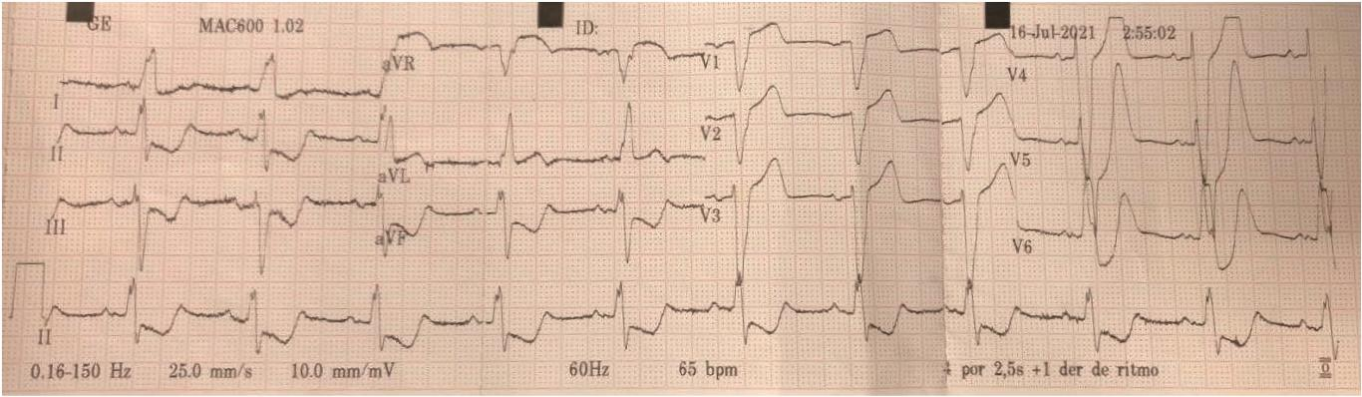


## Case description

A 79-year-old woman with a history of hypertension, managed with losartan and hydrochlorothiazide, was admitted for burning chest pain radiating to the cervical region. She received her first medical care from the ambulance service. The initial ECG showed sinus rhythm, a heart rate of 65 b.p.m., LBBB with negative Sgarbossa criteria, ascending depression at the J point, and peaked T-waves in V4–V6 (‘De Winter’ pattern). Initially, the patient was treated with 300 mg of aspirin (AAS) and 600 mg of clopidogrel and then transferred to the hospital. Upon admission, she continued to experience persistent chest pain and received intravenous nitroglycerine. She was in stable general condition, haemodynamically stable, and without signs of acute heart failure. Initial laboratory tests revealed elevated troponin levels: >500 000 pg/mL (normal value < 15.6 pg/mL) and glucose level of 134 mg/dL (normal range: 70–99 mg/dL). The complete blood count, renal function, electrolytes, and thyroid function levels were within normal ranges. Given the suspicion of acute myocardial infarction, the patient was taken to the haemodynamics laboratory immediately. Cardiac catheterization revealed thrombotic occlusion of the proximal segment of the anterior descending artery (LAD), and aspiration thrombectomy and angioplasty with two drug-eluting stents were performed, achieving angiographic success with a final TIMI 3 flow. A transthoracic echocardiogram showed an ejection fraction of 45% and akinesia in the septal and anterior mid-apical segments. The patient was treated with intravenous nitroglycerine and intravenous furosemide, which were combined with non-invasive mechanical ventilation, resulting in gradual improvement and hospital discharge.

## Discussion

Recognizing the electrocardiographic patterns associated with acute coronary occlusion is essential for diagnosis and immediate reperfusion, thus reducing morbidity and mortality. The ‘De Winter’ pattern, described by Robbert J. de Winter and Niels Verouden, is considered a STEMI equivalent and is associated with occlusion of the LAD. It is represented by an ascending ST-segment depression of 1–3 mm at the J point in leads V1 to V6 and tall, positive, symmetrical T-waves and has an estimated prevalence of 2%.^4^ Although initially described in patients without major conduction abnormalities and once considered a static finding, subsequent studies have suggested that the ‘De Winter’ pattern may represent a dynamic or evolving ischaemic manifestation, potentially progressing to overt ST-segment elevation. Several morphological variants have also been recognized. Nevertheless, its occurrence and diagnostic significance in the presence of LBBB remain poorly characterized, and no clear recommendations exist in current literature regarding its interpretation in this context.^5^ To the best of our knowledge, this is the first reported case of a ‘De Winter’ pattern identified in the setting of LBBB.

Identifying acute myocardial infarction in patients with pre-existing conditions that alter QRS amplitude and width, such as LBBB, represents an additional challenge. In this scenario, the repolarization pattern typically opposes the direction of the QRS complex, generating ST-segment elevation with positive T-waves in leads V1 and V2 and secondary ST-segment depression and T-wave inversion in leads V5 and V6. Sgarbossa *et al.*^6^ proposed criteria for the diagnosis of acute myocardial infarction in the presence of LBBB, which were modified by Smith *et al.*^7^ resulting in a revised rule with greater sensitivity (sensitivity 91% and specificity 90%) compared to the original. The modified Sgarbossa criteria is considered positive when at least one of the three criteria is present: (i) concordant ST-segment elevation ≥ 1 mm in any lead with a positive QRS complex; (ii) concordant ST-segment depression ≥ 1 mm in leads V1, V2, or V3; and (iii) ST-segment to S-wave ratio of ≥0.25 in leads with discordant QRS complexes. In the reported case, despite the absence of diagnostic findings according to the modified Sgarbossa criteria, the presence of a ‘De Winter’ pattern in leads V4 to V6 prompted early coronary angiography, which revealed an occlusive lesion in the proximal LAD.

In conclusion, the case underscores the importance of maintaining a high index of suspicion for the ‘De Winter’ electrocardiographic pattern in the context of acute coronary occlusion and LBBB. Further investigation is warranted to better define its clinical implications and prognostic relevance in this context.

## Lead author biography


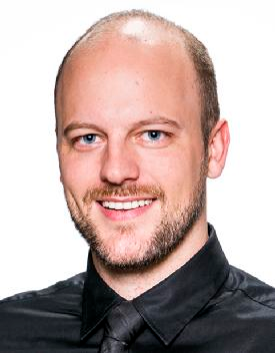


Cardiologist, graduated from the Porto Alegre Clinical Hospital (HCPA). Master’s degree in Cardiovascular Sciences from UFRGS. Professor of Internal Medicine at the Porto Alegre Clinical Hospital and at the Federal University of Rio Grande do Sul (UFRGS).

**Consent:** We hereby declare that we do not have a consent form as the patient is deceased and the patient’s relatives could not be traced. We inform that the patient’s personal data have been duly anonymized, in accordance with the guidelines of the Committee on Publication Ethics (COPE).

## Data Availability

The data underlying this article are available in the article and in its online supplementary material.
